# Improved Data Association of Hypothesis-Based Trackers Using Fast and Robust Object Initialization

**DOI:** 10.3390/s21093146

**Published:** 2021-05-01

**Authors:** Marzieh Dolatabadi, Jos Elfring, René van de Molengraft

**Affiliations:** Control Systems Technology Group, Department of Mechanical Engineering, University of Eindhoven, 5600 MB Eindhoven, The Netherlands; j.elfring@tue.nl (J.E.); M.J.G.v.d.Molengraft@tue.nl (R.v.d.M.)

**Keywords:** initialization, data association, hypothesis tree, tracking, autonomous cars, accuracy

## Abstract

The tracking of Vulnerable Road Users (VRU) is one of the vital tasks of autonomous cars. This includes estimating the positions and velocities of VRUs surrounding a car. To do this, VRU trackers must utilize measurements that are received from sensors. However, even the most accurate VRU trackers are affected by measurement noise, background clutter, and VRUs’ interaction and occlusion. Such uncertainties can cause deviations in sensors’ data association, thereby leading to dangerous situations and potentially even the failure of a tracker. The initialization of a data association depends on various parameters. This paper proposes steps to reveal the trade-offs between stochastic model parameters to improve data association’s accuracy in autonomous cars. The proposed steps can reduce the number of false tracks; besides, it is independent of variations in measurement noise and the number of VRUs. Our initialization can reduce the lag between the first detection and initialization of the VRU trackers. As a proof of concept, the procedure is validated using experiments, simulation data, and the publicly available KITTI dataset. Moreover, we compared our initialization method with the most popular approaches that were found in the literature. The results showed that the tracking precision and accuracy increase to 3.6% with the proposed initialization as compared to the state-of-the-art algorithms in tracking VRU.

## 1. Introduction

The possibility of driving autonomously through an urban environment has been a vision for many years. One of the many challenges of an autonomous vehicle is its safe operation through urban traffic. Therefore, the vehicle needs an accurate description of the environment. Although environment description is a broad topic in autonomous cars, we focus on one part of an environment descriptor. In this work, we propose a probabilistic step to initialize the data association of a hypothesis-based Vulnerable Road User (VRU) tracker.

We consider a situation where multiple pedestrians are crossing the road to illustrate the effect of initialization of the data association. When pedestrians appear in front of a car for the first time, the following steps will happen:

- Records data from its surroundings.
- The detection algorithm detects the positions of pedestrians within data.
- The car estimates the pedestrians’ position and velocity. Then it decides to decrease its speed or brake. At the same time, this detection could be a false positive, leading to unnecessary braking.

Several VRU trackers have been developed in recent years to track VRUs. Some recent studies on this topic are discussed in detail in [1,2,3,4,5,6,7]. These studies track VRUs based on different learning methods and motion prediction models. Although they are useful for tracking VRUs, they have difficulties with the precision or accuracy of their tracks.

The precision of their tracks indicates how well the 2D position and speed of a VRU are estimated. The accuracy of the tracks expresses how many mistakes the tracker made in terms of false positives, the number of tracked VRUs, or the number of VRUs with wrong IDs. The following facts can affect the precision and accuracy of a track:

- Noisy position measurements
- Occlusions

Noisy position measurement includes specification of detectors, environmental situations, such as weather and lighting conditions, and cluttered backgrounds, like trees, traffic signs, and buildings. Image analysis algorithms do not have the same result due to different illumination conditions during the daytime and at night. Occlusions mean objects occlude VRUs entirely or partially, which limits the process of monitoring VRUs. Tracking a VRU in the presence of noisy position measurements and occlusions is a non-trivial task. Therefore, a VRU tracker should satisfy the following requirements:

- Require an algorithm to associate noisy measurements with tracks.
- Contain a state estimation model to predict and describe the movements of each VRU.

**Figure 1:**
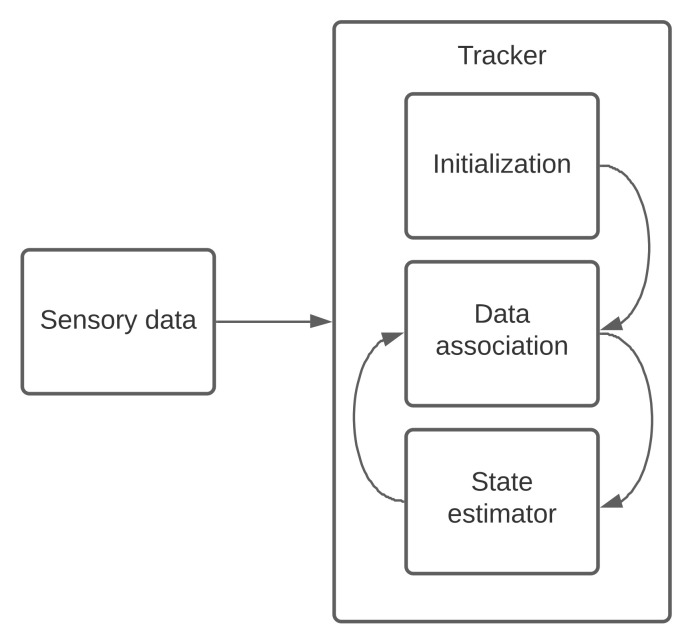
A framework to represent the place of the data association’s initialization.

The data association is a process of matching the measurements of VRUs with a tracker. The measurements can be about the position and the velocity of each VRU. Data association in an urban environment usually suffers from having multiple false alarms and clutters, such as measurement origin uncertainties, besides VRUs generated observations. Therefore, in this situation, data association is faced with many challenges. When data association confirms a track, a tracker’s state estimator will continue to estimate its state vector. After confirming a track, a tracker can predict VRUs trajectories and maintain their identities, regardless of data association errors. It means that incorrect data association leads to potentially catastrophic results. The data association’s initialization primary aim is to provide a guess to decide whether a new filter must be created. The data association should be initialized, confirm or refute a track in a short time in order of a millisecond. All of these facts make the data association complicated. Figure 1 shows the place of the initialization in a hypothesis-based tracker. Our VRU tracker receives detections as input (sensory data), as shown in Figure 1. It also shows a connection between data association and state estimator. It means that, based on a state estimator, our hypotheses are created or updated. In the following sections, we describe how we estimate our state vector and validate our work.

The association of the measurements and process models can underlie the hypothesis tree [8]. This paper incorporates multiple-hypothesis tracking (MHT) with a process model to fulfill the requirements. Solving data association is a pre-requisite for reliable state estimation. Based on how the data association is initialized, most of the existing trackers can be grouped into two categories: model-free-tracking and tracking-by-detection [9]. To initialize a data association, the model-free tracking algorithms require a fixed number of VRUs and tracking-by-detection algorithms need variations in measurements [9]. For instance, Blackman [10] takes five scans of data to initialize its data association. Postponing initialization too long may lead to a late or worse response. Simultaneously, initializing a data association algorithm after a single measurement increases the risk of introducing false positives, which leads to an uncomfortable driving experience.

In this work to initialize a data association for a VRU tracker, we determine a step to initialize a VRU tracker with a probabilistic model. The step helps to choose the correct hypotheses by determining values for parameters within the probabilistic model. In this work, our contributions are as follows:

- We propose a step to find a trade-off between the parameters in a probabilistic model. This step helps data association to reduce the lag between the first detection and track.
- We validate our step’s performance by evaluating it on simulation data, custom-build data, and KITTI raw data.

The remainder of this work organized, as follows: Section 2 discusses literature related to the initialization problem. Section 3 describes MHT to fuse and associate data, and Section 4 presents our VRU tracker. In Section 5, the procedure is validated using different data sets. We conclude and outline future research directions in Section 6.

## 2. Related Work

Among the various studies regarding the initialization of data association of trackers, some of the researchers decide to skip some frames to achieve information regarding the detection [11,12,13,14,15,16]. The skipped frames mean that they remember the detections and start to ‘trust’ a measurement if it appears multiple times. The numbers of skipped frames are varied based on test situations, such as the numbers of VRUs.

Ding et al. [17] use prior knowledge of past detections to initialize the track. They define the probability of the existence of the object based on the past measurements for each object. However, in [18], the authors show that [17] faces more clutters and false tracks when new detections and tracked detections overlap. Leibe et al. [19] utilize several previous frames to initialize a new track. Azim et al. [20] compute the Euclidean distance between the predicted position and new measurements for this purpose. When the distance is less than a certain threshold, they assume that the tracker is initialized. Morimitsu et al. [21] use a fixed number of objects in the first frame. Subsequently, they localize those objects in the subsequent frames. Singh et al. [22] use the global statistics of tracks, linear motion models, and color models to initialize a hypothesis. Schulter et al. [23] localize objects in each frame. Next, they connect those objects to the existing trajectories. Postponing initialization can have a negative impact not only on the quality of a data association, but also on the overall VRU tracker.

As mentioned, a delay in data association makes tracking hard. Different approaches can be used to deal with the data association. For example, nearest neighbor standard filter [24], global nearest neighbor approach [25], joint probabilistic data association [26], MHT, and finite set statistics [27]. Among them, MHT is one of the popular approaches, since it considers data association across multiple input data and multiple hypotheses. MHT grows a tree of hypotheses, based on deterministic branching decisions [28]. To increase the performance of MHT in a VRU tracker, researchers investigate the effects of several parameters, such as tuning VRUs detection, optimizing hypotheses, motion modeling, and initialing tracks. Although initialization is the first step of data associations, it has received less attention than other parameters. In most VRU trackers, the authors utilize similar Poisson-based approaches to achieve prior knowledge and initialize tracks.

In probability theory and statistics, researchers utilize the Poisson distribution of variables to initialize a hypothesis tree. Pan et al. [29] and Moraffah et al. [30] generate the first track by using the average spatial density of new and false-positive object detections in order to address the initialization with Poisson distribution. They use the Poisson distribution for modeling the number of objects in a fixed interval of space or time. Pollard et al. [31] use the Napierian natural logarithm to calculate a score of a track. This score is defined as a probability of the corresponding track. They calculate the first score of each track by utilizing ln of the associated false-positive and correct detection. In another work, Pollard et al. [32] propose a global weight to initialize a track. To do this, they use the track score and a statistical distance between the peak and predict track.

The quick initialization of a data association can allow any trackers to dive into tracking with a lower error, fewer false positives, and the minimal time delay between the first detection and initialization of the VRU tracker. To achieve a quick initialization, we propose steps to reveal the trade-offs between stochastic model parameters. Using the steps, we can minimize the lag between first detection and appearance in a hypothesis-based tracker. In this work, we propose an initialization procedure for a hypothesis-based approach.

We evaluate the tracking of our VRU tracker with the results of RNN, GNN, and RNN-GNN. Recurrent neural network (RNN) is one of the popular methods in multi-target tracking with learned data association methods [33]. Gradient-based neural network (GNN) is also an online time-varying learning-based model for object trajectory [34]. The authors in [34] combine the RNN and GNN (RNN-GNN) to improve traditional online multi-target tracking. In their work, data association depends on the intersection between tracks and detections.

## 3. Multiple Hypothesis Tracker

In this part, we first describe the idea underlying MHT, and then present our reason to select MHT.

### 3.1. Basic Idea

A successful VRU tracker must handle the data association. Data association assigns the correct measurement with the correct track, initializing new tracks and detecting and rejecting measurements. There are different methods for implementing the data association; MHT is a method for solving the data association problem. The reader refers to [8] for a more elaborate explanation of the data association problem and ways to handle it.

MHT generates a tree with several branches. Each branch is a hypothesis, and it forms with different possible associations for each measurement. The principle idea of the MHT is to consider all possible hypotheses in parallel, which means that data association decisions can be deferred until uncertainties on data association are resolved [28].

In this work, each hypothesis $H_{n}^{k}$ contains a list of VRUs, the estimation of their 2D position, and their velocity. Where *n* is hypothesis index n=1,⋯,N and N is the number of hypotheses at time *k*. Furthermore, by applying MHT, it is possible to update a state of VRUs in a probabilistic way with data from the current time.

If no measurement is compatible with one of the existing hypotheses, a new hypothesis or a clutter (a false detection) should be formed. For each hypothesis, the measurement can be explained differently. The VRU tracker fixes the following probabilities for each hypothesis.

- The probability $P_{new}$ of a new VRU. $P_{new}$ indicates the probability that the measurement originates from VRUs not present in the scenes. It means that the data association finds no match between a detected VRU and one specific hypothesis.
- The probability $P_{e}$ of an existing VRU. Different hypotheses have different numbers of VRUs at different locations. Therefore, the number of existing VRUs will vary among hypotheses. $P_{e}$ represents the probability that the measurement originates from the VRUs that are already present in the hypothesis tree.
- The probability $P_{c}$ of a clutter.

The probabilities of the hypotheses being correct is calculated using Bayes theorem.

(1) $$P\left(H_{n}^{k}|Z^{k}\right)=\frac{P\left(Z^{k}|H_{n}^{k},Z^{k-1}\right)P\left(H_{n}^{k}\right)P\left(H_{n}^{k-1}|Z^{k-1}\right)}{P(Z\left(k\right)|Z^{k-1})}$$

where $Z^{k}$ means all of the measurements received up to time *k* and Z(k) means all the measurements received at time *k*. $P(H_{n}^{k}|Z^{k})$ is the posterior probability of the hypothesis with index *n* given all input measurements up to at time *k*, $P(Z^{k}|H_{n}^{k},Z^{k-1})$ is the likelihood, which is a conditional probability for a given measurement at time step *k*. $P(H_{n}^{k})$ is a prior probability at time *k*. $P(H_{n}^{k-1}|Z^{k-1})$ is the posterior probability of the parent hypothesis. Besides, $P(Z\left(k\right)|Z^{k-1})$ shows the normalization term. In this work, the prior probability is a function of the $P_{new},P_{e}$, and $P_{c}$.

Each hypothesis is a parent of multiple hypotheses with the constant ($P_{new},P_{e},P_{c}$) at time k+1. When VRUs are tracked, the probabilities of each hypothesis will be computed, and then the VRU tracker continuously updates the state of VRUs with the next measurements at k+1.

**Figure 2:**
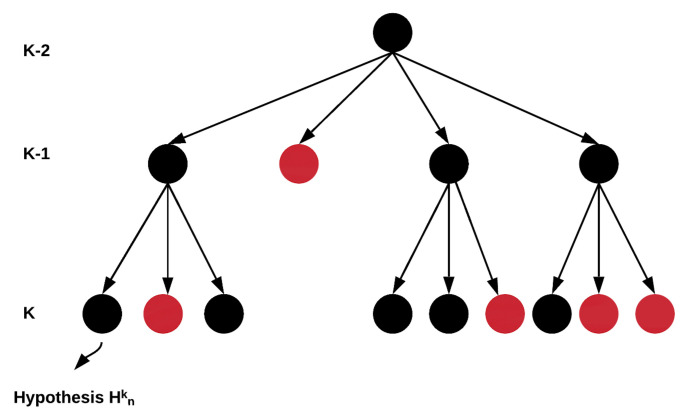
Tree representation of the formed multiple hypotheses. Black dot represents a hypothesis, and a red dot represents a hypothesis that pruned.

Because the VRU tracker has no prior knowledge regarding the number of VRUs, it needs to consider all different hypotheses. Enumerating all of the hypotheses would lead to memory overload fast. Therefore, the growth of the hypothesis tree should be managed by merging or pruning the least probable hypotheses and keeping the most probable one [28]. That way, the tree does not grow exponentially with its depth. Figure 2 gives a schematic of the tree. Each black dot represents a hypothesis and a red dot represents a pruned hypothesis.

As mentioned, each VRU in a hypothesis has three probabilities. These fixed probabilities have a significant role in the management of the tree. Therefore, correct initialization helps to select and keep the most probable hypotheses. For instance, in a case $P_{new}$ is high when compared to the other probabilities, detections quickly lead to new VRUs (according to the most probable hypothesis). As a result, the delay between the first detection and appearance in the VRU tracker’s most likely hypothesis is low.

On the other hand, this also means that more clutters will enter the VRU tracker. Setting $P_{new}$ very low leads to more considerable delays but, at the same time, minimizes the appearance of false positives in the VRU tracker. This paper is about finding a trade-off between these three probabilities for a hypothesis tree.

### 3.2. Reason

Maintaining multiple hypotheses and the ability to correct previous conclusions that are based on new detections are the reasons for choosing the MHT.

## 4. VRU Tracker

To track VRUs, we use an approach introduced [35]. They present a probabilistic environmental description for indoor applications. By changing their state estimator and initialization, we adapt their work to utilize as a VRU tracker for outdoor purposes. We use our previously published work to change their state estimator [36].

### 4.1. State Estimator

We assume that the relative velocity of the VRU with respect to the car is constant over the prediction in order to estimate the 2D position and the velocity of VRUs in a hypothesis. Thus, a Kalman filter with a constant velocity process model is used in our process model. Our state estimator provides the position (m) and velocity (m/s) of pedestrians’ multiple joints related to a detector [36].

**Figure 3:**
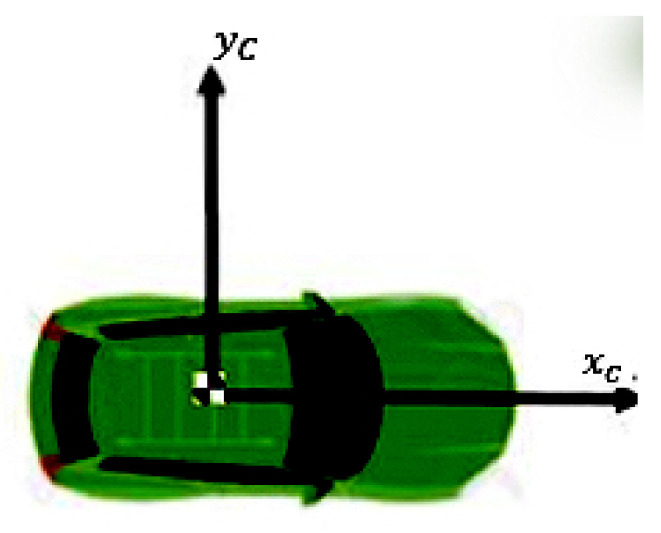
The car frame coordinate system. $x_{c}$ denotes the direction of driving, and $y_{c}$ indicates the side direction of the car.

Figure 3 shows the car frame coordinate system. We denote components of the relative distance of each VRU to the car frame as *x* and *y*. $V_{x}$ and $V_{y}$ also define the velocity of VRU with respect to the car. We discuss our approach to obtain a better understanding of the probabilities and the process model.

- To track VRUs, our VRU tracker collects data from a camera that was mounted on the top of the car. Our measurement contains the 2D position of VRUs. In the next step, the VRU tracker with the MHT framework generates a hypothesis tree in which each branch of the tree is one possible set of data associations. For each hypothesis, the process model estimates the 2D position and the velocity of VRUs. Our VRU tracker, based on the output of the data association and the process model, decides to update the tree or add new branches by receiving a new measurement. Subsequently, it chooses the most probable hypothesis. The probability of person *A* is a new/existing/clutter VRU, and person *B* is a new/existing/clutter VRU.

### 4.2. Performance

We have selected a Multiple Object Tracking Precision (MOTP) and a Multiple Object Tracking Accuracy (MOTA) as performance metrics to evaluate the effects of our initialization on a data association of a VRU tracker [37]. These two types of metrics quantify different relevant aspects. MOTA and MOTP enable us to compare the effect of our initialization on the VRU tracker with other state-of-the-art algorithms. Besides, we indicate that a VRU will be tracked by correct data if we improve data association accuracy. Therefore, we can also improve MOTP.

The difference between the result of our VRU tracker and the ground truth state vector for the most probable hypothesis is (SE). MOTP is an average of SE and the total number of VRUs in the hypothesis c(k). MOTA consists of the number of false detections fp(k), the number of ID switches IDs, and the number of missed VRUs in the most probable hypothesis m(k).

(2) $$MOTA=1-\frac{\sum_{k}\left(m\left(k\right)+fp\left(k\right)+IDs\right)}{\sum_{k}g\left(k\right)}$$

where g(k) is the number of objects present at time k.

(3) $$\begin{array}{cc} MOTP & =\frac{\sum_{j,k}\sqrt{{\left(x_{j}\left(k\right)-xc_{j}\left(k\right)\right)}^{2}+{\left(y_{j}\left(k\right)-yc_{j}\left(k\right)\right)}^{2}}}{\sum_{k}c_{k}}\\ \; & =\frac{SE}{\sum_{k}c\left(k\right)} \end{array}$$

where $xc_{j}\left(k\right)$ and $yc_{j}\left(k\right)$ are the ground truth position of each VRU with respect to the car. We briefly discuss an example to obtain a better understanding of MOTA.

- We assume that our detector recognizes true positive VRUs. Although a VRU tracker correctly receives measurements, it defines one of the VRU as a fp(k). Besides, it tracks more VRUs m(k) that are not available in the input. Therefore, m(k) indicates a mismatch between the number of VRU of a hypothesis and ground truth. Moreover, a VRU tracker may switch the ID of each VRU during tracking that it calls IDs.

This paper is about finding the balance that optimizes the data association performance in terms of MOTA and MOTP. Therefore, we propose sets of values for $P_{new}$, $P_{e}$, and $P_{c}$, which leads to maximizing the metrics in various circumstances. In order to do so, we analyze simulated and real-world data with different numbers of VRUs entering the scene. They are recorded with different vehicles, sensor sets, detection algorithms, and different countries. The results show that there is a possibility to identify sets of values ($P_{new}$, $P_{e}$, $P_{c}$). These sets help us to initialize hypothesis-based data association and maximize the two performance metrics. In general, these sets for each VRU are constant, and we update them in the following situation:

- Whenever we start tracking a new VRU.
- When we have a partial and complete occlusions or the number of VRUs of the current measurement is different from previous ones, we have to re-initialize our data association.

## 5. Results

We define a test case in our simulations to check our data association’s performance in terms of the metrics. In this test case, the car receives the 2D position of the VRUs while the VRUs are crossing a road. In all of the simulations, although we repeat the same test case, we vary the following parameters. It means that these parameters are constant during each test, and we fix them manually before starting each test. By changing each of them, we can produce an effect on the value of the metrics. Moreover, we expect these values to change in real-world applications.

- *R* represents the measurement noise covariance matrix of zero-mean additive Gaussian measurement noise. Based on sensors’ specifications and calibration, we defined a valid range for variation of *R*. Kalman filters use R for estimating the state of VRUs. To estimate *R* in our simulations, we use [38]. We change *R* in our simulation to investigate whether sensors with different amounts of noise require different values ($P_{new},P_{e},P_{c}$).
- The number of moving and standing VRUs. We define a state estimator for each VRU. In each time, our hypothesis tree changes based on the measurements. Moreover, the car may encounter many scenarios in which the number of VRUs and the way they move varies. Therefore, we change this parameter in our simulations to have an optimal data association.
- The probability of new VRUs $P_{new}$, probability of clutter $P_{c}$, and probability of existing VRUs $P_{e}$. As we discussed earlier, these probabilities are used to compute the posterior probabilities of all hypotheses given the measurements. The values of three probabilities have to sum up to one, so, by having two of them, we can compute the third one. The probabilities can be set in different ways, depending on the preferences of the user.

**Figure 4:**
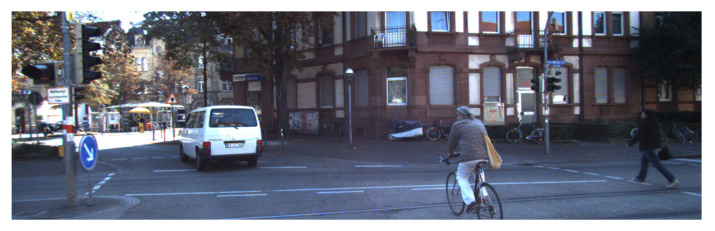
Sequence ‘5’ from the KITTI raw dataset.

We perform real-world experiments on the university campus to validate the sets of values that we identify in simulations. We validate our procedure using KITTI data to benchmark performance on a broader range of scenarios and compare with existing work [39]. KITTI contains the following information at 10 Hz in a city, residential area, campus, and road:

- 3D Velodyne point clouds that we use as ground-truth measurements.
- 3D GPS/IMU data.
- Calibration of sensors working at different rates to obtain ground truth data.
- 3D object track-list labels. Classify objects as a class of pedestrians, cyclists, cars, and trucks.
- Raw and processed color stereo sequences. Figure 4 shows one of the individual benchmarks of KITTI. To collect the data, they equipped a standard station wagon with two high-resolution color and grayscale video cameras [39].

Subsequently, we use all of the results to find an experimental and analytical relation between MOTP, MOTA, and different sets of probabilities. In the last step of the validation, we compare the data association error due to Poisson’s initialization and the proposed sets of probabilities. We used Robot Operating System (ROS) on Linux OS (Ubuntu 16.04) to achieve all the results. We note that the calculations were on an Intel Core i7-6700HQ, CPU 2.6 GHz *×* 8. To log or playback the data, we used ROSBAG. The average size of each ROSBAG file in the simulation was 200 (kB), in the experiment was 50 (MB). For the KITTI benchmark, the average size of each test was 1 (GB).

### 5.1. Simulation

False-negative detections can have a significant effect on the data association and the hypothesis tree to keep or prune a hypothesis. Therefore, in order to find a relation between relevant parameters and the hypothesis tree, in the simulation part, we assume that there is no false-negative detection. We utilize Gaussian noise as an example of our measurement noise.

To optimize the probabilistic models have used within the hypothesis tree, we have taken the following assumptions:

- The sensors deliver the 2D position of VRUs.
- The average VRU walking speed at crosswalks varies between 0 to 1.4 m per second.
- Based on our sensors’ field of view, we can detect a maximum of 20 VRUs.

We have different simulations to investigate whether or not the assumptions influence the values $P_{new},P_{e}$, and $P_{c}$ and the hypothesis tree. Firstly, we run our simulation in an ideal situation without considering the effect of measurement noise. The settings of the simulations vary, as follows:

- Simulate the effect of the number of standing VRUs per area on the VRU tracker. This simulation has been done in an ideal situation.
- Simulate the effect of the walking or/and standing VRUs on the VRU tracker in an ideal situation.
- Repeat the first simulation in a non-ideal situation.
- Repeat the second simulation in a non-ideal situation.

The horizontal axes are $P_{new}$ and the vertical axes are $P_{e}$ in order to read the figures. The summation of the probabilities is one, as we mentioned earlier. For example, when the point in the lower left has $P_{e}$ = 0.2, $P_{new}$ = 0, and, hence, $P_{c}$ = 0.8. Moreover, MOTP is the average overall VRUs/times. In all figures, dark blue means the minimum MOTP, and yellow indicates the maximum MOTP.

**Figure 5:**
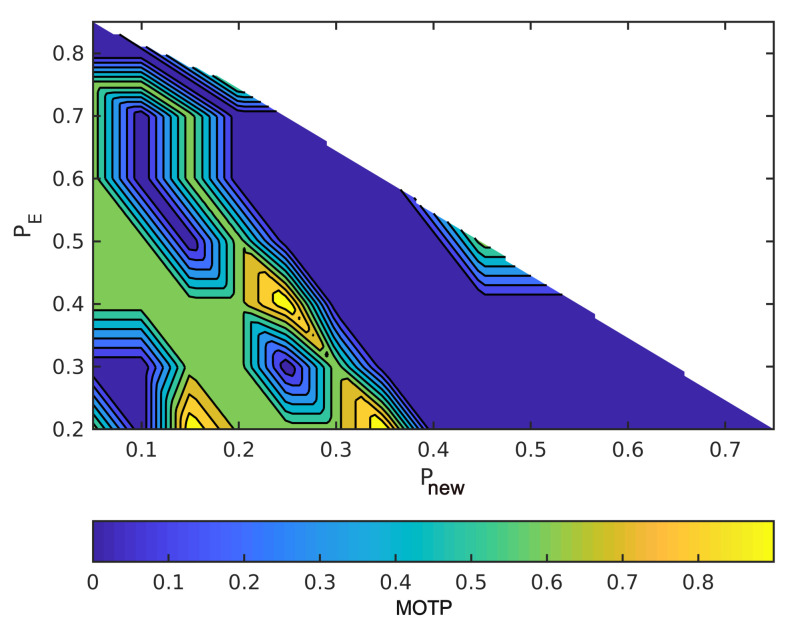
Simulate the effect of different sets of probabilities on MOTP with static VRUs and without covariance noises.

**Figure 6:**
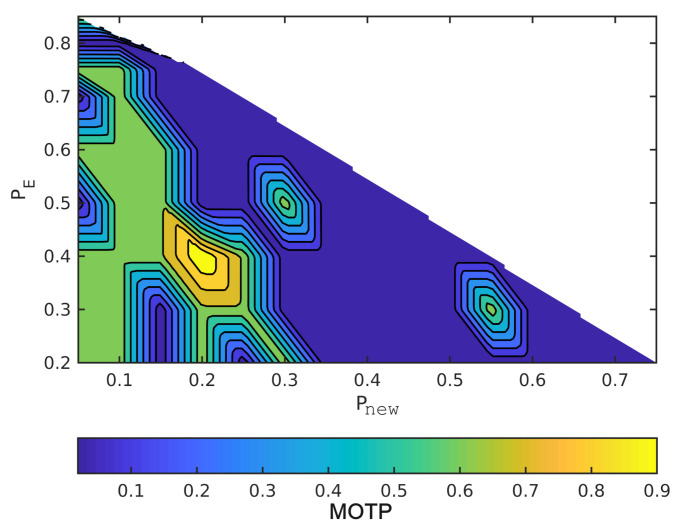
Simulate the effect of different sets of probabilities on MOTP with dynamic VRUs and without covariance noises.

Figure 5 and Figure 6 show varying the probabilities sets to change MOTP. In these two figures, we compute the MOTP of five standing and walking VRUs into an ideal situation. As a result, by changing these probabilities sets, different hypotheses are selected; then, the hypothesis tree delivers different state estimates. Therefore, the hypotheses tree gets the most probable hypothesis faster than the other sets of probabilities by increasing $P_{new}$. Moreover, having the most probable hypothesis shortly after the detection reduces the difference between the ground truth data and process model estimations, especially for walking VRUs.

**Figure 7:**
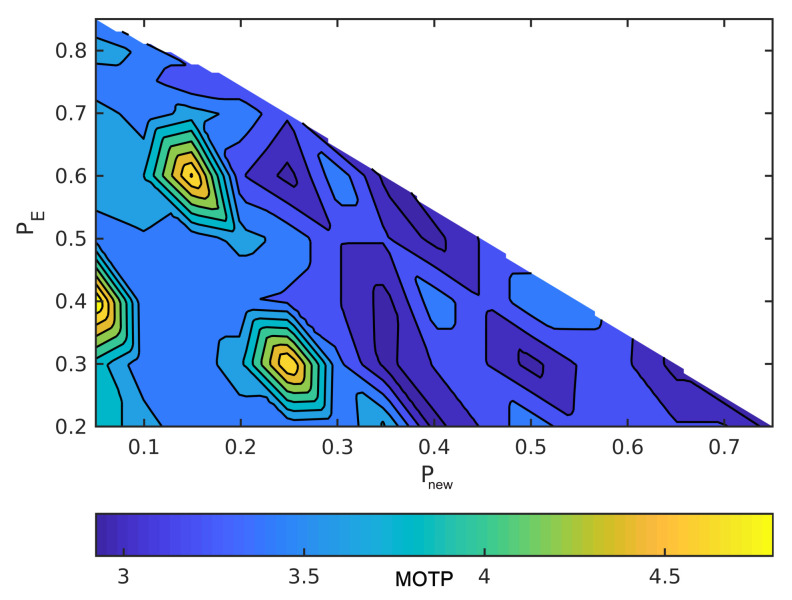
Simulate the effect of different sets of probabilities on MOTP with static VRUs and with one meter measurement noise.

**Figure 8:**
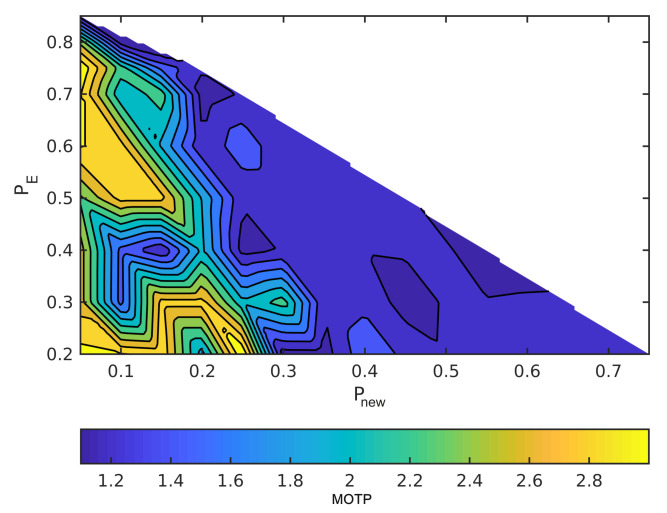
Simulate the effect of different sets of probabilities on MOTP with dynamic VRUs and with one meter measurement noise.

Figure 7 and Figure 8 illustrate the effect of the covariance metric in MOTP. In fact, in these two figures, we repeated the previous simulations using different measurement covariance matrices. Although MOTP increases in Figure 7 and Figure 8, we can achieve different sets of ($P_{new},P_{e},P_{c}$) that generate lower MOTP than the other sets. These sets are almost similar to the first simulation. For instance, if we compare the Figure 8 with Figure 6, we can see a common region in terms of values for $P_{new}$, $P_{e}$, and $P_{c}$, and their MOTP is lower than other part of the region.

Based on the simulations, we identify a common range of values for $P_{new}$, $P_{e}$, and $P_{c}$. The range of values leads to the best trade-off in terms of MOTP.

We repeat the simulations 6400 times in different situations with various speeds, numbers of VRUs, and probabilities for obtaining this region. It means that, in 16 variations of the number of VRUs, walking situation, and measurement noise, we considered a fix $P_{new}$ and varied the $P_{e}$ in a range of 0 to 1 and a step of 0.05. Subsequently, we fixed $P_{e}$ and repeated the same simulation for variation of $P_{new}$ in a range of 0 to 1 by a step of 0.05. Based on the results of all the simulations, Table 1 summarizes the minimum MOTP and its probabilities. Where *W*, *S*, and W−S are walking, standing, and both walking and standing VRUs. We observe that MOTP has the minimum error when the probability of new VRUs bigger than the probability of existing ($P_{new}>P_{e}$) and the probability of clutter ($P_{new}>P_{c}$), as shown in the table. Therefore, a region based on the sets of probabilities is obtained.

The minimum value of MOTP means that the data association works with a lower time delay and skips less measurement than other sets of ($P_{new},P_{e},P_{c}$). It means that the initialization of the data association can help to have a correct guess. Therefore, a correct set of probabilities can decrease the number of false tracks.

### 5.2. Experimental Setup

**Figure 9:**
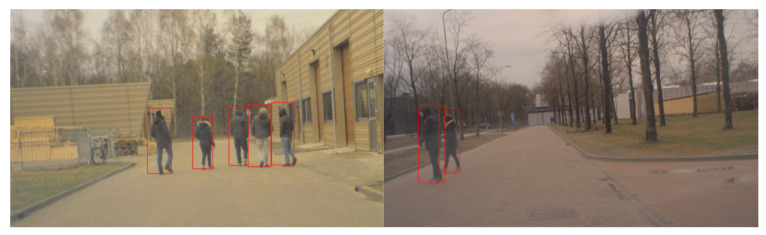
Illustration of our experimental test. The car receives the center of each rectangle as the position of pedestrians.

To experimentally evaluate the range of ($P_{new},P_{e},P_{c}$) procedure, we used a custom-built autonomous car prototype (Toyota Prius, in which sensors and other hardware added). We executed the tests with a vehicle speed of 15 km·h${}^{-1}$ on a university campus. Figure 9 shows the real test situation; the car detects the pedestrians for the first time.

During experiments, we repeated one scenario in 11 different periods of one day in Spring. Investigating the effects of measurement noises and external disturbances, such as weather, light condition, and camera movement on our initialization was our reason for repeating the tests.

We equipped the car with a streaming camera for VRUs detection and a GPS to provide data on its position. In these experiments, we use fast regions with convolutional neural networks (FastR−CNN) to detect VRUs [40]. During the experiments, the pedestrians cross the road, regardless of the presence of the car. After passing an intersection, the car’s camera detected the pedestrians. At the same time, FastR−CNN extracts the pedestrians, and it makes a boundary box around each of them. After that, the VRU tracker receives the center of boundary boxes in the Cartesian coordinate frame as positions of pedestrians at 10 Hz.

**Figure 10:**
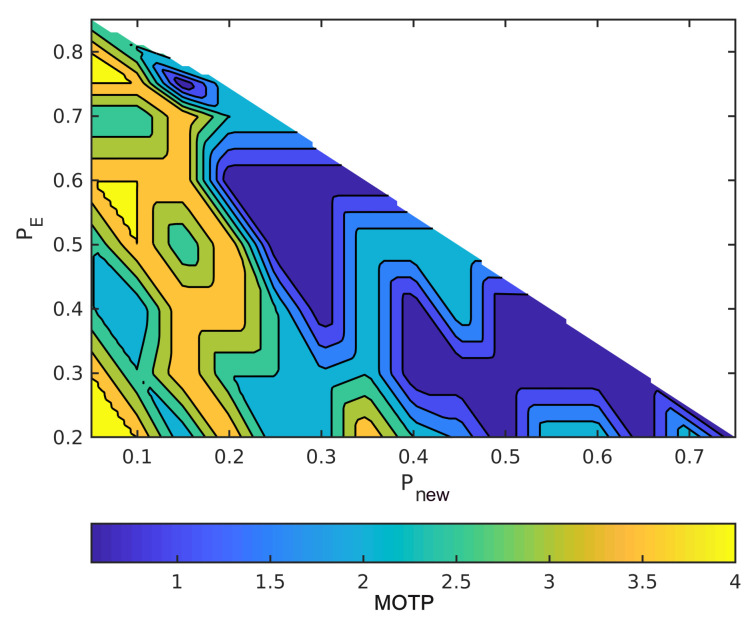
Different sets of probabilities affect MOTP of an experiment test.

Figure 10 illustrates MOTP for different probabilities in an experimental test. Table 2 shows the minimum and maximum values of MOTP when setting the optimal and non-optimal values for $P_{new}$, $P_{e}$, $P_{f}$ in all of the experimental tests. Our reason to compute the maximum value of MOTP is to show the effect of different sets of probabilities on our data association. For instance, in test 1, the VRU tracker estimates the states with 1.8 m error if we define the set from out of the region.

**Figure 11:**
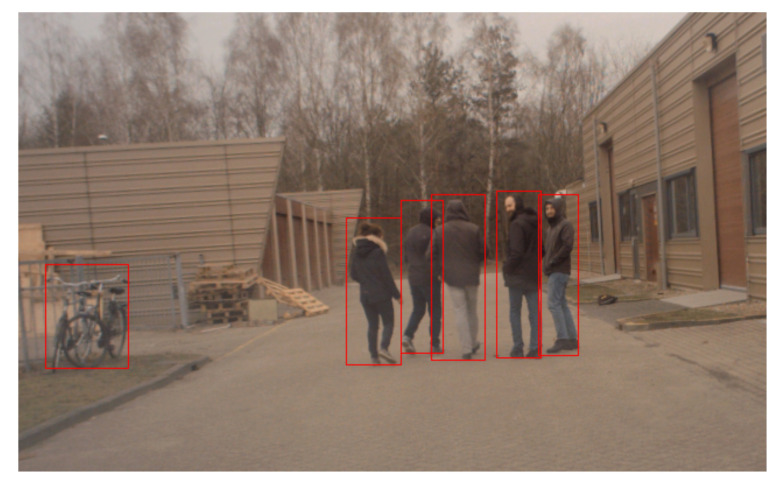
This detection contains a false positive since our detection method detected a bicycle as a VRU. Therefore, our tracker should select a hypothesis that assumes those bicycles are false-positives.

Referring to Figure 10 and Table 2, the region that is mentioned in the previous part is in place. To investigate the effect of the region on MOTA, we compute both MOTA and MOTP in all of the experiments. Based on Table 3, MOTP and MOTA have more reliable results if we select the probabilities from the region. The MOTA values indicate that, although there are false positive detections, our data association does not consider false-positive detections. For example, Figure 11 indicates a real situation that our tracker receives a false-positive detection as a VRU. Based on our initialisation, the data association selects a hypothesis that assumes that the object is a false-positive detection.

In the next step, we investigate the possibility of defining a set of fixed values for $P_{new}$, $P_{e}$, and $P_{c}$, which leads to good performance in all circumstances. Therefore, the following steps have been done:

- We calculate an average of the probabilities in all experiments when we have the minimum errors.
- Subsequently, we compute both metrics based on the average set of probabilities.

Table 4 represents the values of MOTA and MOTP for this set. Although these metrics are lower than Table 3 in the minimum SE, we can have a constant set of probabilities for the initialization. Therefore, there is no need to change the probabilities for different parts of our experiments, since it is inconvenient to dynamically change the probabilities.

The results indicate a significant relationship between the performance of the VRU tracker and the probabilities. Besides, we can find a constant set of probabilities experimentally that can be used in different scenarios. During our experiments, the VRU tracker is associated and tracked up to 0.5 (*s*) faster when we used the constant probabilities.

### 5.3. KITTI Raw Data

As mentioned before, we verify our VRU tracker on raw data recordings of KITTI. We use sequences of the raw data containing pedestrian and cyclist categories for assessments on the raw KITTI data set. Table 5 reveals, for each sequence, the minimum values of SE.

In Table 6, we show the effect of our initialization on [33]. In the same dataset, we use their state estimator and replace their data association algorithm. Subsequently, we initialize their tracker with our initialization. The first line of the table is the MOTA of [33] that was published in the KITTI website. In the second line, we use a hypothesis-based data association and initialize it based on the best MOTA. In the third line, we initialize the hypothesis-based data association using a set of probabilities that we achieved in the experimental part ($P_{new}=0.5$, $P_{e}=0.3$, $P_{c}=0.2$). We call this set "define set” (DS).

Based on Table 6, our initialization produces the highest number of MOTA. It means that, during the initialization, our data association outperforms the benchmark method in terms of MOTA. Additionally, the table shows that. even by setting constant probabilities for all KITTI sequences, we can perform better than a state-of-the-art algorithm. It means that $P_{new}=0.5$, $P_{e}=0.3$, $P_{c}=0.2$ have an acceptable performance to track VRUs. Moreover, a comparison between our result and state-of-the-art in Table 6 reveals that our initialization procedure can minimize the number of false-positive and miss VRUs. The reasons for having less false-positive and miss VRUs are as follows:

- The VRU tracker can keep multiple hypotheses to rematch measurement with VRUs. It means that, if our VRU tracker receives a false positive detection, it can correct itself after receiving the measurements in the next timestamps.
- Besides, selecting the probabilities based on the region helps the data association to match data without skipping measurements. Therefore, the probability of missing VRUs is low.

The initialization affects the results of the full track. Therefore, there is a possibility to improve the performance metrics in the initialization phase. Based on the outputs of simulations, KITTI, and experiments, for the range of the measurement noise, our data association can be initialized by selecting the probabilities from the region.

### 5.4. Comparison

We compare two different types of data association’s initialization, the Poisson distribution and the proposed initialization. Poisson is one of the most popular distributions in the literature. To compare the two methods, we utilize the Poisson distribution to initialize our VRU tracker. We took the same procedure, as in [29]. This reference gives detail regarding setting the Poisson in a hypothesis tree. Besides, we use data from the experimental test on the campus. Although two methods have the same MOTP after initialization, the MOTP of the proposed procedure is less than Poisson. The reason for this difference is that Poisson should skip some frames to achieve information regarding the average of false and new detection. Meanwhile, our initialization generates the tree and estimates the state of VRUs by choosing the probabilities from the region.

**Figure 12:**
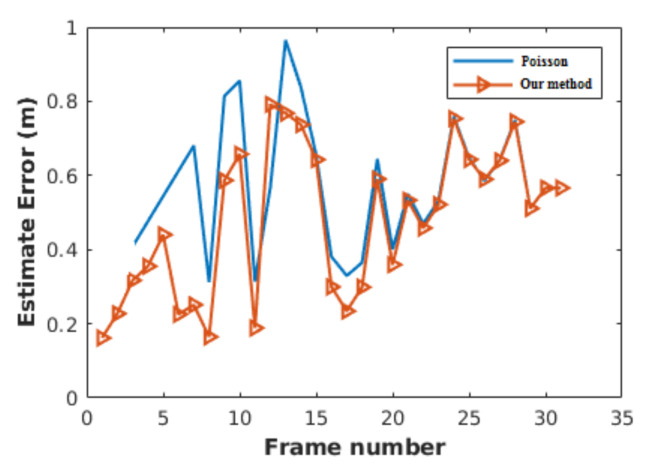
SE of the data association based on the procedure and the Poisson.

SE is a difference between the ground-truth data and the results of a state estimator, as shown in Equation (3). To have a fair comparison between the initialization procedures’ effects, we used the same tracker and test data. The test data were our 11 experiments, and we used [36] for tracking pedestrians. It should be noted that the same tracker means the same data association and the same state estimator. To initialize data association, the Poisson method should wait to collect data. Based on Figure 12, Poisson waits for three frames to collect data and build up confidence, which, here, the waiting time is 0.3 s. As a result ofthe 0.3 s delay in initializing the hypotheses-based data association, the hypothesis tree assumes a part of the measurements in the first 0.3 s is clutter. Therefore, the state estimator could not estimate the states close to the ground-truth data.

In the meantime, based on Figure 12, our trade-off skips only one frame, which means 0.1 s. Therefore, our initialization helps the data association to gain more measurement. Hence, the state estimator can estimate the state vector close to the ground-truth data. As a result of saving 0.2 s, our initialization performs faster than Poisson. Therefore, in Figure 12, initialization with the Poisson approach can lead to 2% less MOTP compared to our initialization. In Table 7, we applied Poisson and our procedure to initialize the data association. Subsequently, we computed the SE of all 11 experiments. Similar to Figure 12, Table 7 shows that our initialization has less error when compared to Poisson.

## 6. Conclusions

His paper presents a collection of probabilistic elements to initialize the data association of VRUs trackers in an urban environment. The initialization of data association can affect the results of the entire track. It means that late initialization could lead to undesired, or even dangerous, situations. Therefore, there is a possibility to improve the performance of trackers by improving their data association. The primary purposes of our initialization are as follows:

- Minimize the delay between first detection and selecting the correct hypothesis.
- Discard false positives from a VRU detection.

We find a trade-off between the parameters in a probabilistic model. We use various simulations, experimental tests, and the KITTI benchmark in different lighting and weather conditions to find the trade-off. We demonstrated that the collection of probabilistic elements are valid for different numbers of VRUs and measurement noise. Moreover, we show that the probabilistic sets help to initialize hypothesis-based data association and maximize the performance metrics.

Using the collection of probabilistic elements, a hypothesis-based tracker can match data without skipping measurements, and a tracker can reduce the probability of missing VRUs. The MHT can compute better probabilities for hypotheses and it is more likely to select the correct one. Therefore, the collection of probabilistic elements have a significant role in the management of a hypothesis tree. Besides, our evaluations show that our approach has a superior performance in the simulation, real-time, and KITTI datasets. The results showed that the tracking precision and accuracy increase up to 3.6% with the proposed initialization as compared to the state-of-the-art algorithms in tracking VRUs.

Multiple group tracking is also challenging in the field of autonomous cars. In future work, we plan to investigate the effect of the initialization procedure on a data association of multiple group tracking. Moreover, in this work, our objects were limited to pedestrians and cyclists. Therefore, in future work, we will also consider the effects of other objects, such as vehicles. 

## Figures and Tables

**Table 1:** Probabilities sets that cause the minimum MOTP for each scenario.

N/VRU	W/S	Noise (m)	Min MOTP (m)	$P_{\mathrm{new}}$	$P_{e}$	$P_{c}$
1	W	0	0.00	0.65	0.2	0.15
2	W	0	0.03	0.45	0.35	0.2
5	W-S	0	0.00	0.4	0.35	0.25
10	W-S	0	0.00	0.45	0.4	0.15
20	W-S	0	0.08	0.45	0.3	0.25
1	S	0	0.00	0.7	0.2	0.1
5	W-S	0	0.00	0.5	0.3	0.2
10	W-S	0	0.80	0.5	0.3	0.2
1	W	1	0.04	0.5	0.3	0.2
2	W	1	0.06	0.4	0.35	0.25
5	W-S	1	0.80	0.45	0.3	0.25
10	W-S	1	0.18	0.45	0.25	0.35
20	W-S	1	0.25	0.5	0.3	0.2
5	S	1	0.40	0.5	0.3	0.2
5	W-S	1	0.80	0.45	0.3	0.25
10	S	1	0.18	0.45	0.25	0.3

**Table 2:** Minimum and maximum of MOTP for each experiment.

No.	Min SE (m)	$P_{n}$	$P_{e}$	$P_{c}$	Max SE (m)	$P_{n}$	$P_{e}$	$P_{c}$
1	0.05	0.6	0.25	0.15	1.80	0.3	0.2	0.5
2	0.16	0.4	0.35	0.25	3.12	0.25	0.3	0.25
3	0.13	0.5	0.3	0.2	2.74	0.1	0.5	0.4
4	0.01	0.6	0.25	0.15	1.55	0.05	0.2	0.75
5	0.14	0.4	0.35	0.25	2.75	0.25	0.25	0.5
6	0.12	0.4	0.3	0.3	2.71	0.1	0.25	0.65
7	0.11	0.65	0.3	0.05	1.42	0.05	0.2	0.75
8	0.09	0.6	0.15	0.25	1.60	0.05	0.2	0.75
9	0.77	0.5	0.35	0.15	3.03	0.05	0.55	0.4
10	0.138	0.6	0.3	0.1	2.10	0.1	0.75	0.15
11	0.01	0.45	0.3	0.25	1.57	0.05	0.2	0.75

**Table 3:** Average of the minimum and maximum MOTP and MOTA for all the experiments.

MOTP Max SE	MOTA Max SE	MOTP Min SE	MOTA Min SE
60%	66%	91%	89%

**Table 4:** The average MOTP and MOTA in a set of probabilities.

$P_{n}$	$P_{e}$	$P_{c}$	MOTP	MOTA
0.5	0.3	0.2	84%	78%

**Table 5:** Minimum SE for each sequences.

No.	Minimum Error (m)	$P_{\mathrm{new}}$	$P_{e}$	$P_{f}$
5	0.023	0.75	0.2	0.05
9	0.27	0.5	0.3	0.2
11	0.06	0.6	0.25	0.15
60	0.10	0.5	0.3	0.2
59	0.06	0.6	0.25	0.15

**Table 6:** Compare our work with a state-of-the-art algorithm on KITTI raw dataset.

Name	MOTA	${\mathrm{Ours}}_{\min}$	${\mathrm{Ours}}_{\mathrm{DS}}$
[33]	72.1%	73.2%	72.6%
[41]	57.61%	57.93%	57.67%
[42]	53.84%	54%	53.86%

**Table 7:** SE of two different initialization procedures.

No.	Minimum SE Our Method (m)	Minimum SE Poisson (m)
1	0.05	0.14
2	0.16	0.25
3	0.13	0.14
4	0.01	0.08
5	0.14	0.16
6	0.12	0.13
7	0.11	0.19
8	0.09	0.20
9	0.77	0.81
10	0.14	0.20
11	0.01	0.03

## Data Availability

The data that support the findings of this work are openly available in https://zenodo.org/communities/autopilot.
